# GATA3-positivity and T follicular helper phenotype in transformed mycosis fungoides with leukemic involvement and prominent folliculotropism: a case report

**DOI:** 10.3389/fonc.2026.1890945

**Published:** 2026-07-27

**Authors:** Gioia Di Stefano, Vieri Grandi, Virginia Alba Colantuono, Chiara Frattini, Benedetta Peruzzi, Roberto Caporale, Nicola Pimpinelli, Raffaella Santi

**Affiliations:** 1 Histopathology and Molecular Diagnostics, Careggi University Hospital, Florence, Italy; 2 Section of Dermatology, Department of Health Sciences, University of Florence, Florence, Italy; 3 Flow Cytometry and Immunology Diagnostic Unit, Careggi University Hospital, Florence, Italy; 4 Pathology Section, Department of Health Sciences, University of Florence, Florence, Italy

**Keywords:** folliculotropism, GATA3, mycosis fungoides, primary cutaneous T-cell lymphomas, Sezary syndrome, T follicular helper

## Abstract

Mycosis fungoides (MF) shows a broad clinical spectrum, ranging from indolent disease to aggressive variants with poor outcomes. T Follicular Helper (TFH) marker expression has been reported in a subset of MF, with uncertain prognostic significance, while GATA3 has emerged as a potential adverse prognostic marker in MF and nodal T-cell lymphomas. Herein, we report a case of MF investigated through sequential histopathological and phenotypic analyses, including extended TFH markers and GATA3 immunohistochemistry. Initially diagnosed as conventional MF, the disease evolved after 3 years into MF with large cell transformation and leukemic involvement, characterized by prominent folliculotropism, a TFH phenotype, and diffuse GATA3 expression. Retrospective analysis of the initial biopsy identified a minor TFH/GATA3-positive neoplastic population (10%) already present at onset, supporting a model of progressive phenotypic plasticity evolving over time. This case underscores the biological heterogeneity of MF, suggesting that a TFH phenotype and GATA3 expression may help identify patients with a more aggressive disease course, potentially amenable to mogamulizumab-based targeted immunotherapy. Incorporating extended TFH markers and GATA3 into routine immunohistochemical assessment could refine risk stratification and inform treatment decisions.

## 1 Introduction

Primary cutaneous lymphomas constitute the second most frequent extranodal lymphomas, after gastrointestinal lymphomas (1, 2). Among these entities, mycosis fungoides (MF) and Sézary syndrome (SS) are the most common, accounting for 50% of all cutaneous T cell lymphomas (CTCLs) (1–3). SS represents approximately 2-3% of all primary cutaneous lymphomas and is characterized by the triad of erythroderma, generalized lymphadenopathy, and clonal atypical cerebriform T cells (Sézary cells) in the skin, lymph nodes, and peripheral blood (1–3). SS typically follows an aggressive clinical course. In contrast, MF exhibits a broad spectrum of clinical presentations and prognostic outcomes, ranging from indolent disease with a favorable prognosis to highly aggressive variants. This marked clinical and histopathological heterogeneity contributes significantly to the diagnostic challenges posed by MF. Prognostic factors associated with aggressive MF have been extensively studied, and patient survival is largely stage-dependent. Advanced disease stages (IIB and higher), including SS, are associated with poor outcomes, with a median overall survival (OS) of less than five years (OS: 4.7 years for stages IIB and IIIA, 3.4yrs for IIIB, 3.8yrs for IVA1, 2.1yrs for IVA2, and 1.4yrs for IVB) (4, 5). One of the histopathological manifestations of disease progression in MF is large-cell transformation (LCT), defined as the transformation of neoplastic small lymphocytes into a clonally identical large-cell phenotype. LCT occurs in 20-55% of patients with advanced MF and is associated with an unfavorable prognosis (6). Consistently, the prospective PROCLIPI (PROspective International Cutaneous Lymphoma Prognostic Index) study identified LCT, stage IV disease, age >60 years, and elevated lactate dehydrogenase levels as independent prognostic markers of reduced survival in MF (7). Although a subset of MF cases, accounting for up to 25%, may progress to SS (8), the diagnosis of SS should be strictly reserved for patients presenting with *de novo* erythroderma. The International Society for Cutaneous Lymphomas (ISCL) recommends that patients with classic MF who subsequently develop erythroderma and peripheral blood involvement be classified as “SS preceded by MF” or “secondary erythrodermic CTCL” (3, 9). In previous studies, MF has been reported to express T-follicular helper (TFH) cell markers in a subset of cases (10–13). As none of these markers is entirely specific for TFH cells, the expression of at least three among PD-1, CXCL13, ICOS, BCL6, and CD10 has been suggested to support a TFH phenotype (14). The prognostic significance of TFH-type MF remains uncertain, as this phenotype has been described both in indolent CTCLs and in clinically aggressive variants (15, 16). However, recent studies have identified a subset of intertriginous MF cases with a TFH phenotype characterized by an aggressive clinical course and progression to SS (12, 17). Herein, we report a case of MF with LCT, leukemic involvement, and prominent folliculotropism, exhibiting a GATA3-positive TFH cell phenotype, arising in the setting of previously diagnosed classical MF.

## 2 Case description

**Figure 1:**
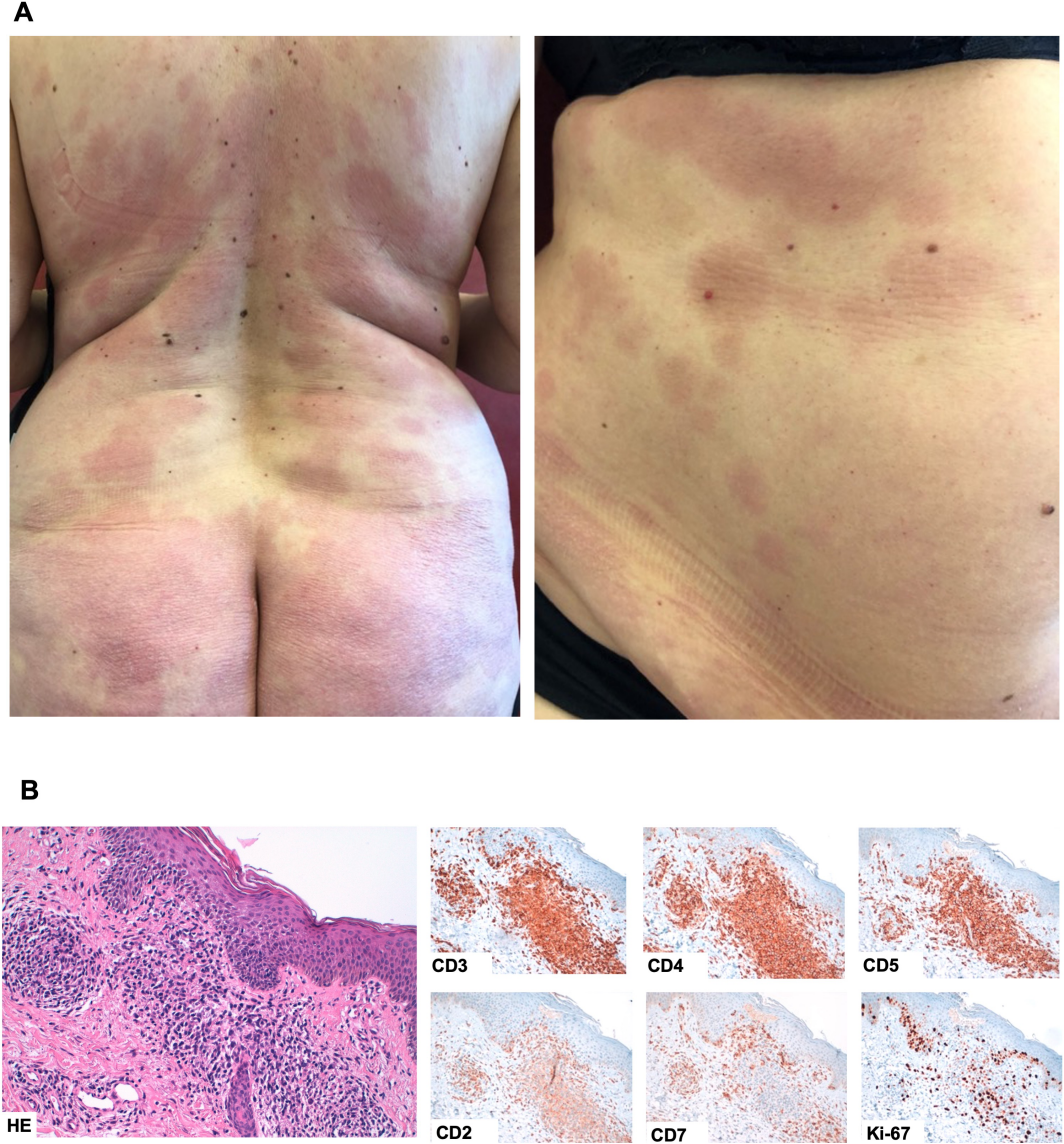
Initial clinical and histopathological findings. **(A)** Diffuse erythematous, mildly scaly patches involving the entire body surface. **(B)** Hematoxylin-eosin (HE) staining showing a perivascular lymphoid infiltrate of small- to medium-sized lymphocytes with focal epidermotropism (original magnification x20). Immunohistochemistry demonstrated a T-cell phenotype: CD3+, CD4+, CD5+, weak CD2 expression, and partial loss of CD7. Ki-67 index was approximately 10–20% (original magnification 40x).

**Figure 2:**
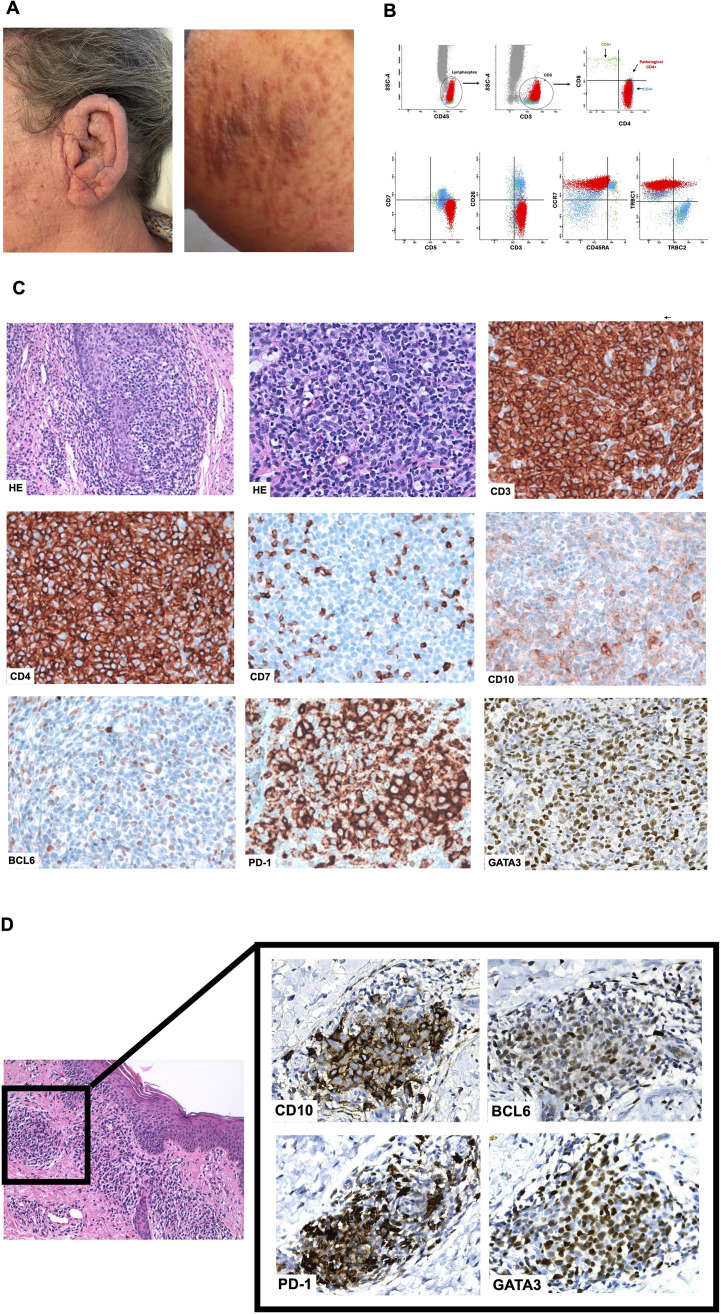
Clinical, histopathological, and immunophenotypic re-evaluation. **(A)** Whitish to skin-colored papules coalescing into plaques with marked involvement of the auricular and frontal regions, consistent with leonine facies, and diffuse erythematous papules on the remaining body surface, suggestive of folliculotropism. **(B)** Peripheral blood flow cytometry showing an aberrant clonal CD4^+^ T-cell population (70.9% of total cells; 1.92 × 10^9^/L) with Sézary-like features CD3+, CD4+, CD5+/++, CD7+ dim, CD26-, CD2+, HLA-DR-, CD56-, CD16-, CCR7+, CD45RA-, TCRα/β+, TCRγ/δ- and unimodal TRBC1 (clone JOVI-1) expression. **(C)** Hematoxylin–eosin (HE) staining revealed a dense, full-thickness dermal perifollicular infiltrate with follicular epithelial invasion (original magnification ×20). The infiltrate was composed of medium-sized lymphoid cells with a significant large-cell component (>25%) (original magnification ×40). Immunohistochemical evaluation demonstrated expression of CD3 and CD4, loss of CD7, and expression of T follicular helper (TFH) markers CD10 and BCL6, PD-1 positivity, and GATA3 expression (original magnification ×40). **(D)** Histopathological re-evaluation of the initial biopsy displayed a subset of perivascular lymphoid cells on HE (original magnification ×20) expressing TFH markers CD10, BCL6, PD-1, and GATA3, indicating the presence of this T-cell subset from disease onset (original magnification ×40).

A 52-year-old woman presented with a 4-year history of widespread erythematous-desquamative patches (**Figure 1A**). On suspicion of MF, incisional biopsies of the left arm and thigh were performed. Both specimens showed similar histopathological features, with a superficial perivascular infiltrate in the papillary dermis composed of small-to-medium-sized atypical lymphoid cells with hyperchromatic, irregular nuclei, and a clear perinuclear halo exhibiting focal epidermotropism but no evidence of folliculotropism. Immunohistochemical analysis demonstrated a T-cell phenotype, with positivity for CD3, CD4, and CD5, weak CD2 expression, and partial loss of CD7. The proliferation index (Ki-67) was approximately 10-20% (**Figure 1B**). T-cell receptor (TCR) gene rearrangement analysis performed according to the BIOMED protocol (18) demonstrated monoclonal rearrangements of both TCR-beta and TCR-gamma genes. These histopathological and molecular findings corroborated the clinical suspicion, leading to a diagnosis of MF. The patient was classified as stage IB disease (19), with skin involvement affecting more than 10% of the body surface area (T2), no clinical or radiological evidence of lymph node or visceral involvement (N0, M0), and no blood involvement (B0). The patient initially received skin-directed therapies (narrowband UVB and PUVA), achieving only a transient response, followed by relapse. Subsequent escalation included SDTs combined with acitretin and later with bexarotene and methotrexate due to inadequate disease control. Three years later, clinical follow-up revealed whitish and skin-colored papules coalescing into plaques, causing marked distortion of the bilateral retroauricular and frontal regions (leonine facies), along with diffuse erythematous folliculotropic papules on the rest of the body (**Figure 2A**). Flow cytometric analysis of peripheral blood revealed an aberrant clonal T-cell population accounting for 70.9% of total cells (1.92x10^9^/L), CD3+, CD4+, CD5+/++, CD7+ dim, CD26-, CD2+, HLA-DR-, CD56-, CD16-, CCR7+, CD45RA-, TCRα/β+, TCRγ/δ- and unimodal TRBC1 (clone JOVI-1) expression (**Figure 2B**). These findings were suggestive of a circulating aberrant T-cell population with Sézary-like immunophenotypic features. A newly biopsy performed in the retroauricular region, showed an atypical lymphoid infiltrate with prominent folliculotropism, absence of epidermotropism, and a mixture of medium-sized haloed cells and large atypical cells (>25%), sometimes with prominent nucleoli, in an eosinophil-rich background (**Figure 2C**). Immunohistochemical analysis demonstrated a T-cell phenotype, with positivity for CD3, CD4, and CD5, weak CD2 expression, and a medium-high proliferation index (Ki-67 approximately 40%). CD30 expression was detected in less than 5% of the neoplastic population. Notably, the large-cell component showed expression of TFH markers, including PD-1, CD10, and BCL6, while CXCL13 and ICOS were negative (**Figure 2C**). The neoplastic cells lacked CD8, CD7, TIA-1, Granzyme B, and CD56 expression, with negative EBV EBER *in situ* hybridization. T-cell receptor (TCR) beta and gamma gene rearrangement analysis was attempted; however, no evaluable results were obtained due to DNA degradation. Given these atypical features, skin biopsies obtained three years earlier were reviewed to assess whether TFH marker expression observed at disease progression was already detectable at initial presentation. On re-examination, a small perivascular subset (approximately 10%) of small- to medium-sized lymphoid cells, lacking distinctive morphological features compared with the surrounding population, showed expression of CD10, BCL6, PD-1, and GATA3, and were negative for CXCL13 and ICOS (**Figure 2D**). Overall, these findings established a diagnosis of MF with LCT and leukemic involvement, characterized by prominent folliculotropism, GATA3 expression, and a TFH cell phenotype, representing progression from previously diagnosed classical MF. Disease restaging revealed multiple clinically abnormal lymphadenopathies, predominantly in the axillary and inguinal regions, with nodes measuring over 2 cm. The patient received mogamulizumab, with good tolerability and sustained clinical benefit. Following the transformation to SS, a structured follow-up was maintained in accordance with European Organization for Research and Treatment of Cancer (EORTC) and ISCL guidelines. Clinical evaluations every 3–6 months showed a very good partial response (VGPR), with residual cutaneous lesions limited to occasional facial patches (mSWAT score of 1), managed with topical therapies and low-dose systemic corticosteroids as needed, without treatment escalation. At 24 months post-diagnosis, hematologic responses were stable, with no signs of progression, as confirmed by regular assessments. Recent physical examinations indicated no palpable lymphadenopathy, reflecting good disease control.

## 3 Discussion

MF represents the most frequent subtype of CTCLs and usually follows an indolent clinical course. However, in a minority of cases, MF may progress to more aggressive lymphoproliferative disorders, including MF LCT and SS-MF. In the present report, we describe a case of MF with LCT and leukemic involvement, showing prominent folliculotropism, and a GATA3-positive TFH phenotype, occurring in a patient with a prior diagnosis of conventional MF. The co-occurrence of these features further emphasizes the biological and clinical heterogeneity of advanced MF, highlighting the overlap and close relationship between MF and SS. The availability of multiple biopsies across different disease stages also provides a unique opportunity to investigate rare but diagnostically and biologically relevant CTCL features, potentially contributing to a better understanding of histopathological factors associated with a more aggressive disease course. Folliculotropism is a well-recognized feature of MF and a defining characteristic of one of its variants; it has been described only rarely in SS (20–22). Occasional reports have described folliculotropism in patients with SS or MF/SS, suggesting that this feature may represent a dynamic characteristic acquired during disease progression rather than a fixed clinicopathologic pattern (21, 22). To our knowledge, progression from non-folliculotropic MF to MF with LCT, leukemic involvement, prominent folliculotropism, and facies leonina has not been previously reported. Most published cases of folliculotropic SS correspond to primary SS, whereas documentation of secondary SS developing after previously non-folliculotropic MF remains extremely limited (21, 22). Previous studies have shown that patients with folliculotropic involvement may also develop leukemic disease and are therefore associated with a poorer prognosis (21). In the present case, the large-cell component of the MF mostly exhibited a TFH phenotype. TFH marker expression has been previously described in MF (10, 11, 13) and in SS (23). Similarly to MF, SS is phenotypically heterogeneous and exhibits a TFH phenotype, with studies indicating that Sézary cells display characteristics of stem cell memory, central memory, effector memory, and transitional memory T-cell subsets (23–26). Notably, retrospective evaluation of the initial biopsy revealed a subpopulation of medium-sized neoplastic cells with a partial TFH immunophenotype, expressing CD10 and BCL6 and showing strong PD-1 positivity. This phenotype, confined to a subset of tumor cells, is indicative of early intratumoral T-cell heterogeneity. Nevertheless, identification of a TFH phenotype in cutaneous T-cell proliferations poses significant diagnostic challenges, particularly because it raises the differential diagnosis of angioimmunoblastic T-cell lymphoma (AITL) involving the skin. In our patient, this diagnosis was excluded based on EBER *in situ* hybridization negativity and the primary cutaneous onset of the disease, with lymphadenopathy developing only at disease progression. In light of growing evidence supporting the role of transcription factors in T-cell lymphomas, we further evaluated GATA3 expression, a key regulator increasingly implicated in both nodal and extranodal T-cell lymphomas (27, 28). Retrospective evaluation of the initial biopsy revealed GATA3 expression within the same subset of neoplastic cells expressing TFH markers, suggesting that the GATA3-positive TFH population was present from disease onset and may represent a biologically more aggressive disease that drives subsequent progression. The co-expression of TFH markers and GATA3 in the neoplastic cells of the present case is biologically intriguing. Under normal physiological conditions, *BCL6* directly represses *GATA3* transcription to enforce TFH commitment (29); nevertheless, this mutual exclusivity may not be absolute: IL-6, secreted by antigen-presenting cells during T-cell priming, has been shown to restrain the development of GATA3-expressing TFH cells, suggesting that in conditions of reduced IL-6 signaling, a GATA3-positive TFH subset may emerge (30). Consistent with this, a subset of AITL (15%), the prototypical TFH-derived lymphoma, can harbor a *GATA3* molecular profile (31). The mechanisms underlying this co-expression in our case and its functional consequences remain to be elucidated. Additionally, our case raises important issues regarding the prognostic significance of TFH phenotype and GATA3 expression in MF. Several reports have described TFH marker expression in advanced MF and transformed cases (10, 11); however, data regarding its prognostic impact remain conflicting. As previously demonstrated, PD-1 expression is significantly increased in SS and is more frequently observed in tumor-stage MF than in patch/plaque-stage disease (32), suggesting a potential association with disease progression, as also observed in our case. In line with previous anecdotal reports (12, 17), our findings support the hypothesis that TFH marker expression may characterize biologically aggressive subpopulations. However, this interpretation is limited by the fact that these markers are not routinely assessed in clinical practice. Regarding GATA3, this transcription factor, together with T-bet, orchestrates Th2 and Th1 differentiation, respectively. MF is known to undergo immune microenvironment remodeling during progression, shifting from a Th1-dominant to a Th2-dominant profile (33, 34), a shift in which GATA3 plays a central role. Thus, Th2 polarization may also converge biologically with the PTCL-GATA3 (Th2-like) molecular subtype (35, 36). Several studies have demonstrated that GATA3 expression correlates with poor prognosis in MF and nodal T-cell lymphomas (27, 28). Immunohistochemical evidence indicates that the disease component responsible for progression and transformation corresponds to the GATA3-expressing TFH population. GATA3 expression alone has been strongly associated with poor prognosis (28). However, we observed an excellent clinical response to mogamulizumab, a CCR4-targeting biologic therapy. This observation is consistent with evidence suggesting that such treatment may be particularly effective in patients exhibiting a Th2-skewed phenotype, characterized by increased expression of both GATA3 and CCR4 (27, 28). The main limitation of this study was the inability to determine clonal relatedness between the initial and subsequent biopsy specimens at the molecular level, as TCR clonality analysis could not be evaluated on the second biopsy due to DNA degradation. In conclusion, we report a case of MF with LCT, prominent folliculotropism, and leukemic involvement characterized by a GATA3-positive TFH phenotype, occurring in a patient with a previous diagnosis of classical MF. Our findings reinforce the concept of phenotypic plasticity and heterogeneity in CTCL, highlighting TFH differentiation and GATA3 expression as potential prognostic markers and predictors of response to mogamulizumab therapy. These data support the potential utility of routinely integrating extended TFH panels and GATA3 immunostaining into diagnostic workflows for CTCL.

## Data Availability

The original contributions presented in the study are included in the article, further inquiries can be directed to the corresponding author.
